# Integrating Mitochondrial Biology into Innovative Cell Therapies for Neurodegenerative Diseases

**DOI:** 10.3390/brainsci14090899

**Published:** 2024-09-05

**Authors:** Adaleiz Ore, James M. Angelastro, Cecilia Giulivi

**Affiliations:** 1 Department of Molecular Biosciences, School of Veterinary Medicine, University of California Davis, Davis, CA 95616, USA; adore@ucdavis.edu (A.O.); jmangelastro@ucdavis.edu (J.M.A.); 2 Department of Chemical Engineering, School of Engineering, Case Western Reserve University, Cleveland, OH 44106, USA; 3 University of California Medical Investigations of Neurodevelopmental Disorders Institute (MIND Institute), University of California Health, Sacramento, CA 95817, USA

**Keywords:** mitochondrial medicine, cell therapy, neurodegenerative diseases, stem cells, exosomes, extracellular vesicles, mitochondrial dysfunction, ethical concerns

## Abstract

The role of mitochondria in neurodegenerative diseases is crucial, and recent developments have highlighted its significance in cell therapy. Mitochondrial dysfunction has been implicated in various neurodegenerative disorders, including Alzheimer’s, Parkinson’s, amyotrophic lateral sclerosis, and Huntington’s diseases. Understanding the impact of mitochondrial biology on these conditions can provide valuable insights for developing targeted cell therapies. This mini-review refocuses on mitochondria and emphasizes the potential of therapies leveraging mesenchymal stem cells, embryonic stem cells, induced pluripotent stem cells, stem cell–derived secretions, and extracellular vesicles. Mesenchymal stem cell–mediated mitochondria transfer is highlighted for restoring mitochondrial health in cells with dysfunctional mitochondria. Additionally, attention is paid to gene-editing techniques such as mito-CRISPR, mitoTALENs, mito-ZNFs, and DdCBEs to ensure the safety and efficacy of stem cell treatments. Challenges and future directions are also discussed, including the possible tumorigenic effects of stem cells, off-target effects, disease targeting, immune rejection, and ethical issues.

## 1. Introduction

Neurodegenerative diseases represent a significant challenge in modern medicine due to their progressive nature and the limited efficacy of existing treatments [1]. Previous strategies have focused on various approaches, including pharmaceutical interventions, neurorehabilitation strategies, and surgical procedures [2,3,4]. However, the progressive nature of these diseases and the limited efficacy of existing treatments have prompted researchers to explore new avenues, such as cell therapy.

Cell therapy, a promising avenue in regenerative medicine, has garnered substantial attention for its potential in treating neurodegenerative disorders. Addressing mitochondrial dysfunction in the context of cell therapy for neurodegenerative diseases represents an essential avenue for potential therapeutic interventions. Mitochondria play a vital role in regulating cellular energy metabolism, calcium homeostasis, and apoptosis, and their impairment has been implicated in the pathogenesis of various neurodegenerative disorders such as Parkinson’s disease, Alzheimer’s disease, and amyotrophic lateral sclerosis [5,6,7,8,9]. Disruptions in mitochondria dynamics, including an imbalance between mitochondrial fission and fusion rates, are critical to mitochondrial dysfunction and result in impaired function and abnormal morphology. In the brain, a highly energy-demanding organ, this may lead to synaptic degradation [10,11,12].

Recognizing the significance of mitochondrial dysfunction in neurodegenerative disorders, researchers are exploring the potential of cell therapies to address this dysfunction, potentially offering a pathway to slowing the progression of these debilitating diseases.

## 2. Advances in Cell Therapy

### 2.1. Stem Cell–Based Therapies

With their unique capacity for self-renewal and differentiation, stem cells offer a versatile platform for cell-based therapies in neurodegenerative diseases. The efficacy of the use of various stem cell types, including embryonic stem cells (ESCs), induced pluripotent stem cells (iPSCs), and mesenchymal stem cells (MSCs), has been demonstrated in various preclinical and clinical settings [13,14], with numerous phase 1 trials in progress [15,16,17,18]. ESCs and iPSCs hold great potential for generating diverse neural cell types, offering a scalable source for transplantation [19]. However, ethical considerations and tumorigenicity remain significant hurdles [20,21,22]. Nonetheless, advancements in differentiation protocols and genetic manipulation techniques have enhanced the safety and efficacy of pluripotent stem cell–derived therapies [23,24].

**Figure 1:**
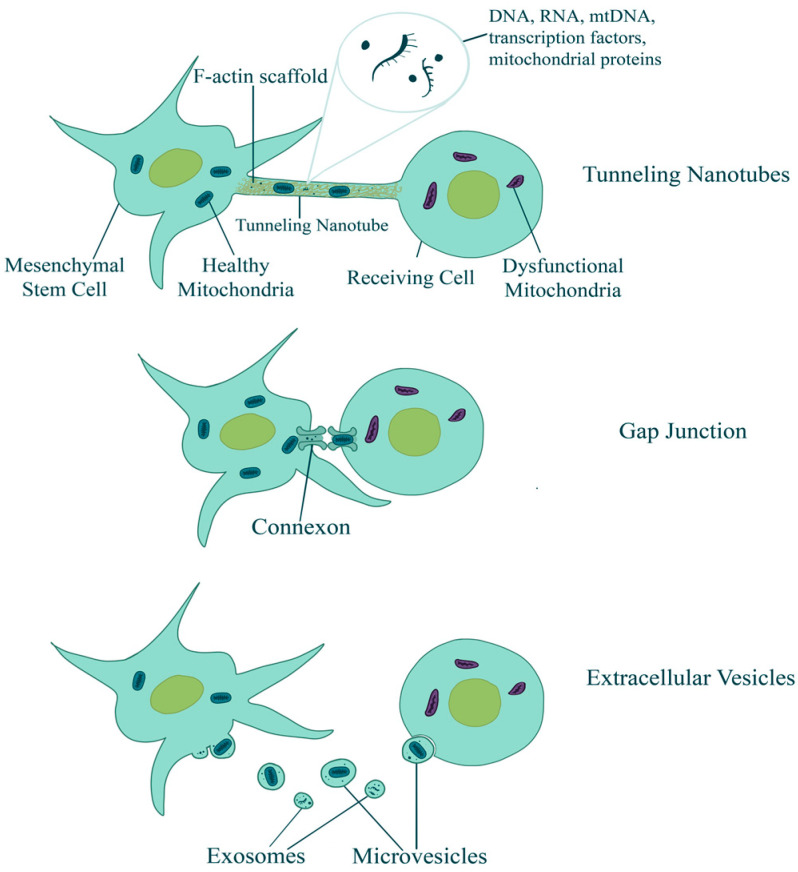
Schematic representation of mechanisms behind MSC-mediated mitochondrial transfer. MSCs can transfer healthy mitochondria to distressed cells, particularly those with dysfunctional mitochondria. Three main mechanisms for the intercellular transfer of whole mitochondria have been identified: tunneling nanotubes (TNTs), gap junctions, and certain types of extracellular vesicles. TNTs are membrane tubes that act as a bridge between more distant cells. Gap junctions are intercellular channels that form direct connections between nearby cells. EVs, lipid-based particles secreted by cells, may contain whole mitochondria or smaller mitochondrial fragments. It is important to note that not all EVs are large enough to contain functional mitochondria.

MSCs, derived from bone marrow and adipose tissues, exhibit immunomodulatory, anti-inflammatory, and neuroprotective properties and tropism for cells in distress, making them attractive candidates for neurodegenerative disease therapy [25,26]. Furthermore, studies have shown that MSCs and iPSCs can transfer mitochondria to cells with dysfunctional mitochondria by forming tunneling nanotubes (TNTs), gap junctions, and extracellular vesicles ([27,28,29]; Figure 1). The receiving cells (*in vivo* and *in vitro* models; Table 1) have shown recovery of function, increased levels of aerobic respiration, and a greater survival rate.

It is unsurprising that MSCs positively affect neurological diseases by improving mitochondrial function when we consider that their principal effects include influencing immune cells, producing antimicrobial peptides, and transferring mitochondria to damaged cells. Preclinical studies suggest that priming MSCs before exposure to harmful environments can enhance these actions [36]. A key benefit of MSCs is their ability to improve mitochondria function in damaged tissues by enhancing mitochondrial quality control (MQC).

In cell therapy, MSCs and NSCs can influence mitochondrial biogenesis and fusion–fission dynamics in neighboring cells during cell therapy through different mechanisms, including paracrine signaling, release of extracellular vesicles, direct mitochondrial transfer, and immunomodulatory effects. These mechanisms contribute to the therapeutic potential of these stem cells by enhancing mitochondrial function, reducing oxidative stress, and promoting tissue repair [37].

Regarding paracrine signaling and the release of bioactive molecules that can influence mitochondrial dynamics in neighboring cells, MSCs secrete factors like vascular endothelial growth factor (VEGF), insulin-like growth factor (IGF-1), and hepatocyte growth factor (HGF), which can enhance mitochondrial biogenesis in neighboring cells [38,39,40]. These factors activate signaling pathways, such as the PI3K/Akt pathway, leading to the activation of PGC-1α and other transcriptional regulators of mitochondrial biogenesis in recipient cells. MSCs also secrete anti-inflammatory cytokines (e.g., IL-10, TGF-β; [40]) that can modulate mitochondrial dynamics by reducing oxidative stress and stabilizing mitochondrial networks [41]. This can result in enhanced mitochondrial fusion and a more interconnected mitochondrial network in damaged or stressed cells, crucial for cellular recovery and function. It has been shown that MSCs inhibit fibrosis by releasing stanniocalcin-1 (STC-1). This protein acts in multiple ways (e.g., reducing the secretion of collagen by fibroblasts and TGFβ output by endothelial cells) but is also relevant to mitochondria; uncoupling mitochondrial respiration via the induction of uncoupling protein 2 alleviates oxidative stress [42].

Neural stem cells (NSCs), derived from central nervous system tissues or differentiated from pluripotent cells, also hold the potential for cell-based therapies. NSC transplantation in mice with Alzheimer’s disease has been found to reduce symptoms and correct abnormal mitochondrial morphology by regulating mitochondrial fission, fusion factors, and specific mitochondrial proteins [43]. NSCs can release neurotrophic factors such as brain-derived neurotrophic factor (BDNF) and glial cell line–derived neurotrophic factor (GDNF) [44,45]. These factors can stimulate mitochondrial biogenesis in nearby neurons and glial cells, promoting neural repair and functional recovery. For example, BDNF can activate the CREB pathway, leading to increased expression of PGC-1α and other regulators of mitochondrial biogenesis [45]. It can be envisioned that NSCs can also modulate mitochondrial dynamics in neighboring cells by releasing signaling molecules that reduce apoptosis, promote mitochondrial fusion, and activate the SIRT1 pathway [44,46]. For example, NSCs secrete factors inhibiting DRP1 activity, thereby decreasing mitochondrial fission and promoting and maintaining a healthy mitochondrial network in stressed or injured neural cells.

MSC-derived EVs can transfer mitochondria or mitochondrial components (e.g., mtDNA, proteins involved in mitochondrial biogenesis) to recipient cells. This transfer can enhance mitochondrial function and biogenesis in damaged cells, particularly in cardiac repair and neuroprotection. EVs from MSCs can also carry miRNAs and proteins that regulate mitochondrial dynamics. For example, MSC-derived EVs improved acute renal ischemia-reperfusion injury by inhibiting mitochondrial fission through miR-30 [47]. Mitochondria from MSC-derived EVs, after being endocytosed by recipient cells, are degraded through the lysosomal pathway while others fuse with the recipient’s mitochondrial network accompanied by the regulation of crucial fusion–fission proteins such as MFN1/2 and OPA1 [48]. After mitochondrial transfer and incorporation of donor mitochondria into recipient cells, outcomes linked to mitochondrial function, such as ATP-linked oxygen consumption, mitochondrial mass, and volume, increase [49]. Since extracellular vesicles can carry mitochondrial components of different sizes, ranging from 500 to 900 nm for entire mitochondrial units to 50–100 nm carrying small mitochondrial microdomains [50,51], and NSC-derived EVs have been reported to carry neuroprotective miRNAs and proteins that can promote mitochondrial biogenesis in neighboring neurons and glial cells [52,53], EVs may also influence mitochondrial fusion–fission dynamics by delivering specific proteins and miRNAs that regulate these processes. For instance, they may provide OPA1 or MFN2 to promote fusion or inhibit DRP1 to prevent excessive fission, helping maintain mitochondrial integrity in stressed neurons.

In addition to secreting bioactive molecules and EVs, MSCs have been shown to transfer healthy mitochondria to neighboring, damaged cells within the neurodegeneration process directly through TNTs, restoring mitochondrial function, enhancing energy production, and activating biogenesis pathways in the recipient cells [54,55]. MIRO1, a calcium-sensitive protein domain of mitochondrial Rho-GTPase, plays a major role in intercellular mitochondria transfer from MSCs to cells [56] with other proteins (e.g., KIF5, TRAK1/2, Myo10/19), enhancing the mitochondrial movement inside the nanotubes [30]. Therefore, it is conceivable that during neurodegeneration, the mitochondrial transfer from healthy neurons to stressed ones is prevented, thereby jeopardizing their rescue, or that neurodegeneration-mediated stress impairs (instead of triggering [57]) the transfer of damaged mitochondria. It is conceivable that the healthy, transferred mitochondria can integrate into the recipient cell’s mitochondrial network, influencing fusion and fission dynamics, improving metabolic efficiency, and enhancing cell survival in damaged tissues. Through TNTs, MSCs can accept damaged mitochondria from tumor cells [58] and increase tumor resistance [54,59,60].

Additionally, the immunomodulatory effect of MSCs and NSCs on the damaged tissue microenvironment can potentially impact mitochondrial dynamics in neighboring cells. For instance, by reducing proinflammatory cytokines like TNF-α and IL-1β, MSCs and NSCs can decrease the oxidative stress in the tissue microenvironment [41,61]. This reduction in oxidative stress helps preserve mitochondrial integrity and promotes a balance between mitochondrial fission and fusion in the affected cells. However, some of these factors (TNF-*α*, GM-CSF, MCP-1, IL-17, IL-1*β*, IL-12p70, and CD30L) are closely related to NF-*κ*B signaling pathway, which is involved in the regulation of TNT formation and mitochondrial transfer [62,63,64]. Thus, modulating these factors may also undermine the TNT formation, thereby inhibiting or downregulating the TNT-mediated mitochondrial transfer.

The investigation of the underlying causes of mitochondrial dysfunction in neurodegeneration may provide more insights into this issue. Understanding how MSCs affect MQC could lead to new treatments for neurological conditions. Focusing on transplanting MSC-derived mitochondria to damaged tissues may be a promising new therapeutic approach [65]. However, most of the mechanisms underlying these processes are still uncertain. For instance, MSCs enhanced the expression of the heme oxygenase-1 (HO-1) enzyme, commonly associated with anti-inflammation and immunoregulation, in damaged cells [66]. Overexpression of HO-1 protects against oxidative stress and upregulates several proteins involved in mitochondrial biogenesis, fission–fusion processes, and MQC, including NRF1, NRF2, PGC1α, and TFAM [67]. Reduced expression or impairment of these proteins has also been implicated in several neurodegenerative diseases, including Huntington’s [68], Alzheimer’s [69], Parkinson’s diseases [70], and amyotrophic lateral sclerosis [71], whereas its overexpression reduces detrimental symptoms [72]. Therefore, it is possible that by inducing HO-1 expression (via an unknown mechanism), MSCs can restore mitochondria health in recipient cells. It is essential to be aware that overexpression of HO-1 in tumor cells has also been found to increase aggressiveness and resistance to therapy and may be one of the causes of stem cell–related tumorigenesis [73].

Specific factors determining the transfer of healthy mitochondria via TNTs, gap junctions, and extracellular vesicles are currently being studied. The precise mechanisms by which cells initiate, selectively package, and transfer these mitochondrial components still need to be fully understood. This research is essential for advancing our understanding of cellular biology and mitochondrial studies. Additionally, the selective packaging and transfer of functional mitochondria or smaller mitochondrial fragments via extracellular vesicles is an active research area [74]. Due to the significant role mitochondrial dysfunction plays in many neurodegenerative diseases, further investigation into the mechanisms and causes of initiation of MSC-mediated mitochondrial transfers is warranted to ensure the safety and efficacy of this approach (e.g., [75]). Preclinical and clinical trials using MSCs have had promising results in restoring mitochondrial function [76,77,78]. In vitro MSC therapy reduced oxidative stress and improved mitochondrial function in Alzheimer’s through mitochondrial transfer and MSC’s neuroprotective secretion [31]. Direct injection of mitochondria also improved symptoms and mitochondria function in rat models of Parkinson’s, supporting the role of mitochondrial transfer in MSC-based therapies [32]. Clinical trials employing MSC transplantation showed promising outcomes, with improvements in motor function and neurologic symptoms in patients with Parkinson’s disease and amyotrophic lateral sclerosis [79,80]. Further research in this area is needed to fully understand the mechanisms of MSC-related amelioration and determine the role of mitochondrial transfer and neurotrophic factor secretion.

### 2.2. Gene-Modified Cell Therapies

Gene-editing technologies, notably CRISPR-Cas9, have revolutionized cell therapy by enabling precise modification of cellular genomes. Engineered stem cells with enhanced survival, neurotrophic factor secretion, hypo-immunogenicity, or resistance to disease pathology have demonstrated improved therapeutic outcomes in preclinical models of neurodegenerative disorders [81,82,83,84,85,86]. Future studies identifying the mechanisms behind stem cell tropism for distressed cells and tumors, combined with genetic modification, can improve targeting methods and reduce mitochondrial transfer to tumor cells.

Mitochondrial gene-editing techniques hold potential for neurodegenerative treatment. They would allow for correcting disease-causing mutations in patient-derived iPSCs, overcoming challenges associated with immune rejection, and paving the way for personalized cell-based therapies [87]. Genome editing of patient-derived iPSCs also allows for the generation of isogenic models, enabling treatment optimization before administration to a patient [88]. While challenges such as off-target effects and delivery methods persist, ongoing research efforts with MSCs and hematopoietic stem cells have shown progress in optimizing gene-editing strategies for safe and effective clinical translation [89,90].

**Figure 2:**
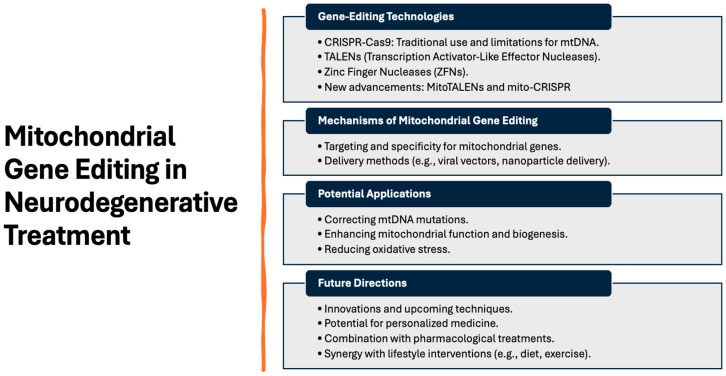
Overview of mitochondrial gene-editing strategies with the potential use for neurodegeneration and other mtDNA-linked diseases.

Recently, several advancements have been made in gene-editing techniques in mitochondria ([87]; Figure 2). MitoTALENs (mitochondrial transcription activator-like effector nucleases) are specialized gene-editing tools derived from the TALEN technology designed to target and modify mitochondrial DNA (mtDNA). They consist of a DNA-binding domain derived from transcription activator-like effectors (TALEs) and a nuclease domain, usually FokI, which introduces double-strand breaks in DNA. The DNA-binding domain of MitoTALENs can be engineered to recognize specific DNA sequences within the mitochondrial genome. This allows for precise targeting of mtDNA mutations. Once bound to the target sequence, the FokI nuclease induces a double-strand break, prompting cellular repair mechanisms to either fix the mutation or remove damaged DNA. By selectively targeting and cleaving mtDNA with pathogenic mutations, MitoTALENs can reduce the proportion of harmful mutations in mitochondrial genomes.

Several studies have reported using MitoTALENs to correct specific mtDNA mutations in cellular function and disease [91,92,93,94,95,96,97,98,99,100,101,102,103]. However, the efficient delivery of MitoTALENs to mitochondria within cells is a significant challenge. In addition, minimizing unintended cuts in nontargeted regions of mtDNA is crucial to ensure safety and efficacy, especially in a genome with no intron–exon structure.

Mito-CRISPR (mitochondrial clustered regularly interspaced short palindromic repeats) adapts the CRISPR-Cas9 system for editing mtDNA. Traditional CRISPR-Cas9 systems cannot naturally target mtDNA, but advancements have enabled this possibility. The CRISPR system utilizes a guide RNA (gRNA) to direct the Cas9 nuclease to a specific DNA sequence. For mito-CRISPR, the gRNA is modified to target sequences within the mitochondrial genome. The Cas9 nuclease creates double-strand breaks at the target site, leading to repair processes that can correct mutations or delete defective mtDNA. This technique can be used to edit mitochondrial genes directly, offering potential treatments for diseases caused by mtDNA pathogenic mutations [104,105,106,107]. This technique also has challenges, such as ensuring that the CRISPR components (gRNA and Cas9) are efficiently transported into mitochondria, achieving high specificity to avoid unintended genetic alterations in the mtDNA or nDNA.

By leveraging the strengths of MitoTALENs and mito-CRISPR, current research aims to develop effective treatments for mitochondrial-related diseases, providing hope for conditions currently lacking effective therapies. MitoTALENs and mito-CRISPR are designed to offer high specificity for mtDNA sequences with different mechanisms for targeting and cleavage. The efficiency of delivery and precise targeting are critical challenges for both techniques, considering the extra complexity that poses the presence of heteroplasmic mtDNA mutations [91,92,94,98,99,100,101,102,107,108,109]. Improvements in delivery vectors and targeting strategies are essential for their successful application. A key component of mtDNA gene-editing techniques is to recall that (according to the mitochondrial endosymbiosis hypothesis) mitochondria originate from ancient symbiotic bacteria [110]; as such, it is imperative to recognize the unique environment and repair mechanisms that mitochondria have, posing additional challenges for effective gene editing. Both techniques hold promise for correcting mitochondrial DNA mutations that cause neurodegenerative diseases and other mitochondrial disorders. Their development and optimization are crucial for translating these technologies into clinical treatments. Finding reliable delivery methods to transport these gene-editing tools into mitochondria while enhancing the specificity to prevent off-target effects is a key challenge in future directions. Furthermore, rigorous testing in preclinical and clinical settings is needed to ensure safety, efficacy, and ethical considerations.

While we highlighted mito-CRISPR and MitoTALENs due to their recent advancements and applications, we recognize the importance of including other significant technologies [87,111,112,113,114,115]. In this context, mitochondrial zinc finger nucleases (mito-ZFNs) have been an essential tool for targeted mitochondrial DNA modification [116]. Mito-ZFNs are engineered nucleases designed to specifically target and modify mtDNA, offering potential therapeutic avenues for diseases caused by mutations in the mitochondrial genome. The zinc finger nucleases (ZFNs) combine the DNA-binding domain of zinc finger proteins with the DNA-cleaving domain of a restriction endonuclease, typically FokI. When tailored for mitochondrial applications, these nucleases are engineered to include a mitochondrial targeting sequence (MTS) that directs them to the mitochondria, where they can selectively bind to and cleave mtDNA. The specificity of ZFNs comes from the zinc finger domains, which can be engineered to recognize specific DNA sequences. By binding to these sequences, the ZFNs can introduce site-specific double-strand breaks in the mtDNA. These breaks can lead to either the degradation of the mutant mtDNA or the induction of homology-directed repair mechanisms. However, the latter is challenging in mitochondria due to the absence of a robust homologous recombination system. In the context of cell therapy, mito-ZFNs can potentially selectively target and eliminate mutated mtDNA that causes mitochondrial diseases [117,118,119,120]. These diseases are often due to heteroplasmic mutations, where both normal and mutant mtDNA coexist within a cell. Mito-ZFNs can be designed to target the mutant mtDNA specifically, reducing its proportion (heteroplasmy level) and thereby alleviating disease symptoms. This selective degradation can help restore mitochondrial function in cells derived from patients with mitochondrial disorders [120]. In MSC or NSC therapies, mito-ZFNs could enhance the quality and therapeutic efficacy of the transplanted cells. By ensuring that the mtDNA in these cells is free from deleterious mutations, mito-ZFNs could improve the stem cells’ overall mitochondrial function and energy metabolism. This enhancement could be particularly beneficial in therapies for tissues with high-energy demands, such as the brain, heart, or skeletal muscles. Mito-ZFNs could also play a role in MQC in cell therapy applications. By selectively targeting damaged or dysfunctional mtDNA, these nucleases could help maintain a healthier population of mitochondria within the therapeutic cells. This MQC could enhance the longevity and functionality of the transplanted cells, leading to better therapeutic outcomes. Still, challenges remain, such as the efficient delivery of these nucleases into the mitochondria of target cells. Delivery systems, such as viral vectors, liposomes, or nanoparticle-based methods, must be optimized to ensure that mito-ZFNs reach the mitochondria sufficiently to exert their effects. While ZFNs are designed for specificity, there is still a risk of off-target effects, where the nucleases may bind and cleave nontarget sequences in the mtDNA [117]. Such off-target activity could lead to unintended mutations or deletions, complicating the therapeutic application. Therefore, the specificity of mito-ZFNs must be rigorously validated. Finally, unlike nuclear DNA, mitochondria lack an efficient homologous recombination repair pathway, making precise genome editing in the mitochondria more challenging. This limitation means that while mito-ZFNs can effectively induce double-strand breaks, the repair outcomes may be less predictable, primarily resulting in the degradation of the cleaved mtDNA rather than its precise correction.

In addition, base editors, such as the DddA-derived cytosine base editors (DdCBEs), represent a novel and powerful addition to the mitochondrial gene-editing toolkit [97,121,122,123,124,125,126,127,128]. These editors allow for precise, single-base modifications in mtDNA without requiring double-strand breaks, which significantly reduces the risk of off-target effects and has opened new avenues for correcting pathogenic mitochondrial mutations, which opens up new possibilities for treating mitochondrial diseases and improving the efficacy of cell-based therapies [97,121,123,124]. DdCBEs are engineered enzymes that allow for the targeted conversion of cytosine (C) to thymine (T) within mtDNA without the need for double-strand breaks (DSBs). This precision editing is achieved through a combination of several components: 

(1) DddA (DddAtox) is a cytosine deaminase that converts cytosine to uracil (derived from the bacterium *Burkholderia cenocepacia*; [124,125]), which is subsequently read as thymine during DNA replication.

(2) TALE (transcription activator-like effector) domains are custom-designed proteins binding to specific DNA sequences. In DdCBEs, TALE domains guide DddA to the target site within the mtDNA. 

(3) Split-DddA architecture. To prevent off-target deamination and ensure that the editing occurs only at the intended site, the DddA enzyme is split into two inactive halves fused to separate TALE domains. 

When both TALE domains bind adjacent to each other at the target site, the DddA halves reassemble into an active enzyme, allowing for precise cytosine deamination. This design ensures high specificity, allowing for targeted editing of mtDNA with minimal risk of unintended mutations. Because point mutations in mtDNA cause many mitochondrial diseases, DdCBEs can be used to precisely correct these mutations by converting a pathogenic cytosine to thymine [129]. For example, a mutation that changes a codon from a normal amino acid to a pathogenic stop codon can be reversed, restoring the normal function of the mitochondrial protein. This capability is particularly valuable in heteroplasmic conditions, where a mixture of normal and mutant mtDNA exists within the same cell. DdCBEs could selectively edit the mutant mtDNA, reducing the proportion of pathogenic genomes and potentially alleviating the disease. In MSC or NSC therapies, DdCBEs could be applied to edit mtDNA in these stem cells before transplantation, correcting any preexisting mutations that might impair their function or survival. This preediting step could enhance the therapeutic efficacy of stem cells, particularly in tissues with high-energy demands. 

DdCBEs can also introduce specific mutations into mtDNA, allowing researchers to create accurate cell models of mitochondrial diseases [97,130]. These models can be used to study disease mechanisms and to test potential therapies. Such models are invaluable for preclinical testing of cell-based therapies, providing insights into how edited mtDNA behaves in different cellular contexts. As evidenced above for other technologies, DdCBEs challenges include some of these factors. One is the delivery of these editors into the mitochondria of target cells. The mitochondrial targeting sequence (MTS) fused to DdCBEs must be optimized for effective import into mitochondria. Current DdCBEs are primarily focused on C-to-T conversions. However, recent studies expanded the range of base conversions (e.g., adenine to guanine), thereby broadening the scope of mtDNA mutations that can be corrected using base editors [131,132]. Although DdCBEs are designed for high specificity, there is always a risk of off-target effects [133], where the editor might bind and modify unintended sites within the mtDNA. Such off-target activity could introduce deleterious mutations, complicating their use in therapeutic applications. Therefore, rigorous validation of DdCBE specificity is necessary before clinical application. In cells with a mixture of normal and mutant mtDNA (heteroplasmy), editing by DdCBEs may not uniformly affect all copies of the mtDNA. The dynamics of heteroplasmy and genetic drift could influence the long-term outcomes of the editing, potentially leading to the reemergence of the mutant mtDNA. Understanding and controlling these dynamics is crucial for the success of DdCBE-based therapies. Lastly, as with all gene-editing technologies, using DdCBEs in human therapies raises ethical and regulatory questions. These include concerns about the long-term effects of mitochondrial editing, the potential transmission of edited mtDNA to offspring (in the case of germline edits), and the need for stringent oversight to ensure patient safety.

### 2.3. Exosome-Based Therapies

Exosomes, small extracellular vesicles (EVs) secreted by cells, have emerged as potent mediators of intercellular communication and therapeutic cargo delivery [134]. While too small to contain functional mitochondria, exosomes may contain transcription factors, mitochondrial proteins, and partially degraded mitochondrial DNA (mtDNA) [135,136]. The purpose of this cargo and how mtDNA enters the exosome is unclear, but it is theorized that exosomes may play a role in mtDNA degradation [135]. Studies have highlighted mesenchymal stem cell–derived exosomes’ neuroprotective and regenerative effects on dysfunctional mitochondria in various neurodegenerative diseases, offering a noncellular alternative to traditional cell transplantation approaches [137,138]. MSC exosomal treatment for neurocognitive recovery in aged mice showed promising results related to the SIRT1 signaling pathway and increased HO-1 and NFR2 expression [139], likely supporting mitochondria biogenesis. Because the mechanisms of stem cell–related amelioration are not fully understood, much of their protective effects may result from their exosomal secretion [137,140,141].

Exosomes are also being investigated for early diagnosis of Parkinson’s and Alzheimer’s because they contain unique levels of specific mitochondrial proteins and signal factors compared to exosomes from healthy cells [142,143]. Further studies of these biomarkers and exosomal cargo in affected patients may help illuminate possible therapeutic payloads for exosomes as drug delivery carriers. The secretome of healthy NSCs, which contains exosomes and other neurotrophic growth factors, has also been identified for its neuroprotective properties and improved mitochondrial function in models of Parkinson’s [44].

Mitochondria-derived vesicles (MDVs) are similar to exosomes but are secreted by mitochondria and are used for intracellular delivery. Alternatively, MDVs’ cargo may be transferred and secreted from the cell in EVs (Table 2). MDVs can selectively remove damaged or misfolded mitochondrial proteins, transferring those proteins to lysosomes for degradation; they are believed to act as a secondary MQC to mitophagy [144,145]. Dysfunction in the PINK1–Parkin pathway, characteristic of Parkinson’s, may inhibit MDV biogenesis and has been linked to the dysregulation of autophagy [146]. An amyotrophic lateral sclerosis–linked mutation in SOD1 reduced MDV formation and accelerated aging [147]. Conversely, in brain models of various neurological conditions such as Down Syndrome and autism spectrum disorders, there has been a noticeable increase in the frequency of MDVs and their associated protein cargo [148,149,150].

In the context of aging, there is a significant increase in the frequency of MDV generation [149]. Because MDVs are small, membrane-bound structures that bud off from the mitochondria and carry damaged proteins and lipids away for degradation or recycling, this process helps maintain mitochondrial integrity and function by selectively removing damaged components without catabolizing the entire organelle [157]. As organisms age, various cellular processes, including mitochondrial function, deteriorate [158]. In this context, the MDV increase is believed to be a response to the higher levels of mitochondrial damage that occur as cells age. By producing more MDVs, cells can enhance their ability to manage and mitigate mitochondrial dysfunction [159]. This is particularly important in post-mitotic cells like neurons, which are not readily replaced and rely heavily on efficient mitochondrial function for their long-term survival and function [160,161]. These vesicles are hypothesized to be compensatory, mitigating deficiencies in other MQC mechanisms. The increased production of these vesicles and their proteins might help maintain cellular function by clearing damaged mitochondrial components and ensuring the proper distribution of mitochondrial proteins, thus supporting cellular health in compromised mitochondrial quality control [162].

**Figure 3:**
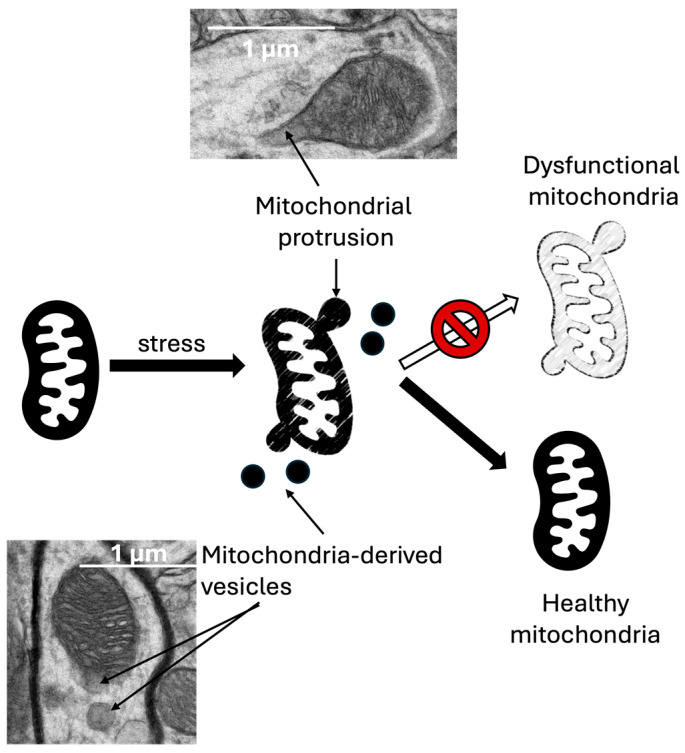
Mitochondria-derived vesicles (MDVs) are small, membrane-bound structures that separate from mitochondria and help maintain cellular balance and respond to stress. They assist in removing damaged mitochondrial components and preserving mitochondrial integrity and function, which are essential for overall cellular health and resilience against stress. MDVs play a significant role in maintaining cellular balance and mitochondrial quality control, which is crucial in neurodegenerative diseases associated with mitochondrial dysfunction and oxidative stress. TEM images (11,000×) were taken from wild-type mouse cortex, identifying mitochondrial protrusions and MDVs. Other experimental details can be found under [165].

An increase in protrusions, budding, and MDV formation has also been reported in neuronal cells under stress, indicating MDVs’ role in reducing reactive oxygen species (ROS)–related damage ([163]; Figure 3). MDVs hold potential both as a diagnosis biomarker and in MQC rescue. Further research on the cargo of MDVs and the pathways that induce their biogenesis could lead to EV-based therapies that increase MQC function and improve mitochondrial dysfunction. Despite this, difficulties in EV, MDV, and exosome characterization are ongoing, and continuing studies are crucial to any future MDV-based therapies [164].

Exosome- and EV-based therapies circumvent many limitations associated with cell engraftment and immunogenicity while providing a means for targeted delivery of bioactive molecules such as microRNAs, mitochondrial proteins, and growth factors [166,167,168]. Despite these benefits, there is still the possibility of an immune response to exosomes containing high amounts of mtDNA, which may induce inflammation similar to that of unpackaged mtDNA [169]. Circulating free mtDNA acts as an antiviral signal, triggering strong leukocyte and cytokine reactions in response to pathogens [170,171]. This response could interfere with neurological functions. Thus, future studies focusing on inflammation because of mtDNA-rich exosomes are crucial to ensure the safety of these techniques [172,173]. Exosomes’ inability to self-replicate prevents any off-target tumor growth associated with cell therapies [174]. They also hold promise for neurological disease treatment due to their unique ability to cross the blood–brain barrier bidirectionally [175]. Trials evaluating exosome therapy in neurodegenerative disorders are underway, with preliminary results suggesting potential benefits in disease modification and symptom alleviation [176,177].

## 3. Challenges and Future Directions

Despite the significant progress in cell therapy for neurodegenerative diseases, several challenges remain to be addressed. These include optimizing cell sourcing, standardizing manufacturing processes, ensuring long-term safety and efficacy, overcoming immune rejection to cell-based therapies or mtDNA and proteins in exosomes, and graft-versus-host responses [178,179,180].

Moreover, the complex pathophysiology of neurodegenerative disorders necessitates multidisciplinary approaches integrating cell-based therapies with gene-editing technologies, pharmacological interventions, neurorehabilitation, and precision medicine strategies. Future research should focus on refining cell delivery methods, enhancing therapeutic targeting and monitoring, and elucidating the mechanisms underlying treatment responses and disease progression. 

## 4. Off-Target Effects

Cell therapy holds immense promise in treating neurodegenerative diseases like Parkinson’s, Alzheimer’s, and Huntington’s diseases; however, like any medical intervention, it has its challenges and potential side effects (Table 3).

Off-target effects on host mitochondria can occur during cell therapy for neurodegenerative diseases. Mitochondria are crucial in producing energy for the cell and are essential for cellular function. However, off-target effects can further damage the host mitochondria, impairing energy production and potential cell dysfunction. This can affect the patient’s overall health and may pose challenges in cell therapy for neurodegenerative diseases. It is an important consideration during the development and implementation of cell-based therapies.

One of the most concerning off-target effects is the potential for transplanted cells to form tumors. This risk arises especially when using pluripotent stem cells, which can differentiate into various cell types. The iPSCs are more tumorigenic due to epigenetic changes that occur during the use of transcription factors during the reprogramming of these cells [181]. Increased risk of tumorigenesis is also associated with the transcription factors used to manufacture these cells [182,183]. Research has shown that the risk of tumor formation for iPSCs varies considerably based on the reprogramming factors used and culturing techniques [184]. These cells might undergo uncontrolled proliferation, leading to the formation of tumors, such as teratomas or carcinomas. In addition, exposure of iPSCs to the microenvironment of preexisting cancer cells could transform iPSCs into cancer stem–like cells [185]. To resolve some of these issues, proto-oncogenes c-Myc and SOX2 were successfully substituted with other less oncogenic transcription factors. Likewise, chemical induction of iPSCs and suicide systems have been employed to eliminate undifferentiated iPSCs and thus reduce the teratoma potential [184,186]. 

Modulating immunogenicity can reduce tumor formation but increases tissue rejection risk [21,187]. Existing tumors may be exacerbated by mitochondria transfer supporting their high metabolic needs; MSC-mediated mitochondria transfer has shown tropism for tumor cells, leading to increased tumor growth and aggressiveness [25,61,188,189]. Potentially attenuating these pro-tumorigenic effects, disrupting GAP43 function in existing tumors could diminish mitochondrial uptake into the tumor cells [190]. Preliminary, noninvasive imaging of the tissue where the MSCs would be placed could identify preexisting tumors before MSC therapy, reducing the risk of tumor enhancement. Likewise, the MSCs could recruit normal cells to adopt a neoplastic phenotype. The role of MSCs in tumor development is complex and subject to ongoing research. Several studies have demonstrated that MSCs can support tumor growth by creating a pro-tumorigenic environment. For instance, MSCs can secrete various factors (e.g., VEGF, TGF-β, and IL-6) that promote tumor cell proliferation, angiogenesis, and metastasis [191,192]. Additionally, MSCs can modulate the immune response to favor tumor growth by suppressing antitumor immune activities and promoting the formation of a supportive tumor microenvironment [191,193]. 

Conversely, some studies have shown that MSCs can inhibit tumor growth under certain conditions. The inhibitory effects of MSCs have been observed in various cancer models, including breast cancer and melanoma, where MSCs induced tumor cell apoptosis and reduced metastasis [194]. This dual role suggests that the impact of MSCs on tumor development may depend on factors such as the source of MSCs, the type of tumor, and the specific conditions of the tumor microenvironment. Based on the current evidence, post-MSC therapy monitoring for early detection of abnormal cell growth is crucial to ensure safety. If abnormal cell growth becomes evident, modified radiographic response assessment in neuro-oncology (mRANO; [195]) could be adapted to record the tumor progression.

**Table 3:** Key ethical, technical, and biological challenges of stem cell therapy.

Stem Cell Types	Advantages	Disadvantages	References
ESCs	Programed to differentiate neural cells without epigenetic interference High proliferation rate	Ethnical issues—destruction of human embryos Tumorigenicity potential Immune rejection	[23,179,181,196,197,198,199]
iPSCs	iPSCs can be derived from any adult tissue without the need for embryos iPSCs can be generated from a patient’s own cells, reducing the risk of immune rejection Highly versatile based on their potential to differentiate onto any cell type	Preprogrammed epigenetics could interfere with differentiated neural phenotype Tumorigenicity potential Issues with efficient reprogramming and stability of iPSCs	[181,184,200,201,202,203,204]
MSCs	Modulate the anti-inflammatory response Low immunogenicity, less rejection Neuroprotective properties Mitochondria transfer Relatively easy large-scale production	Can create a microenvironment that supports tumor cell proliferation and metastasis Limited differentiation capacity (e.g., to neurons) *In vitro* senescence during expansion with potential deleterious effects Heterogenous population affecting consistency and predictability of potential outcomes	[26,54,55,187,191,193,205,206,207,208,209,210]

Tumor cells (i.e., Jurkat cells) have also been shown to transfer damaged mitochondria to MSCs to facilitate their clearance, thereby reducing oxidative stress caused by ROS and increasing tumor cell survival [59]. Because many chemotherapeutic drugs function by increasing ROS levels, this ability may contribute to tumor cells’ chemotherapy resistance [211,212]. It is not definitively documented whether MSCs also uptake dysfunctional mitochondria from noncancerous cells. If this phenomenon is confirmed, it could potentially indicate a protective function of MSCs under normal physiological circumstances. However, it is essential to note that while mitochondrial transfer shows promise in addressing mitochondrial dysfunction in neurodegenerative diseases, there are also potential concerns. For example, mitochondrial transfer to tumor cells results in accelerated growth and increased resistance to chemotherapies, most notably glioma stem cells acquiring MSC mitochondria and developing resistance to temozolomide [61,188,189]. The result is poorer outcomes for patients with gliomas. 

Transplanted cells might trigger immune responses in the recipient’s body, leading to their rejection and prevention of their incorporation into host tissue [198]. This rejection can occur due to human leukocyte antigens (HLAs) differences between the donor and recipient or foreign antigens on the transplanted cells [213]. Immune rejection is less common with autologous iPSCs but may still occur [200]. Exposed mtDNA or mtDNA-containing exosomes may increase the risk of rejection by triggering the immune system and inflammation, causing damage to surrounding tissues and exacerbating the neurodegenerative process [171,173,214]. This inflammatory response can lead to symptoms such as swelling, pain, and dysfunction in the brain. In rare cases, cell therapy has been associated with inflammation and thromboembolisms, which would severely affect the brain [215].

In CNS, and in the context of neurodegenerative diseases, transplanted cells may not integrate properly into the existing neural circuitry, leading to functional deficits or unintended consequences [216,217]. While the primary goal of cell therapy is to restore lost function or halt disease progression, the transplanted cells could inadvertently interfere with normal neural function if they settle in inappropriate locations. Introducing cells into the brain could disrupt the delicate balance of blood flow and vascular function, potentially leading to complications such as hemorrhage, ischemia, or vascular malformations [218]. This could manifest as motor dysfunction, cognitive impairment, or exasperation of existing symptoms [215,216].

Difficulties preventing off-target effects are exasperated by the brain’s heterogeneity and difficulty defining what brain regions should be targeted. Cataloging the diversity of cell types that make up the brain using cell morphology, physiology, transcriptomics data, and time-dependent transcriptional and epigenomic states is essential with cell-based therapies to ensure precise targeting [219,220,221]. Further research into the effects of neurological diseases on different parts of the brain and the most effective potential targets for cell therapies can help prevent many of these off-target effects [222,223].

Many off-target effects of cell therapy may not manifest immediately but could become apparent over the long term. Therefore, long-term monitoring of patients is essential to identify any delayed adverse effects and ensure the safety and efficacy of the treatment [224]. Ongoing research focuses on improving the specificity and safety of cell therapies through strategies such as genetic engineering to reduce immunogenicity, optimization of cell delivery techniques, enhancing cell survival and integration, and refining patient selection criteria [225].

## 5. Ethical Concerns

Cell therapy for neurodegenerative diseases in the context of mitochondria presents various ethical considerations that require careful examination. Mitochondria play a crucial role in cell therapy, producing energy and regulating cell death. Therefore, ethical considerations may arise concerning using mitochondrial manipulation in cell therapies for neurodegenerative diseases. These considerations could include the potential risks and benefits of altering mitochondrial function and the ethical implications of modifying the genetic material within mitochondria. It is essential to carefully assess these ethical considerations to ensure the responsible advancement of cell therapy for neurodegenerative diseases in the context of mitochondria.

Ethical concerns in mitochondrial medicine are an essential aspect to consider due to the innovative nature of this field. One of the primary ethical concerns is using ESCs and iPSCs in mitochondrial medicine. The sourcing of these cells raises ethical questions, particularly regarding the destruction of human embryos in the case of ESCs and the potential for genetic manipulation in the case of iPSCs [22,199]. Furthermore, ethical considerations surround the potential tumorigenicity of pluripotent stem cell–derived therapies. Using these cells in treatments raises concerns about the risk of tumor formation and the long-term implications for patients. Monitoring patients receiving cell therapy over the long term is essential to ensure adequate follow-up care while addressing any unforeseen adverse effects that may emerge over time [226].

Another ethical consideration in mitochondrial medicine is mitochondria transfer, mainly when this transfer occurs to preexisting tumor cells or tumor precursors. This raises concerns about the potential for accelerated tumor growth and increased resistance to therapy, which could have significant ethical implications for patient safety and the overall effectiveness of mitochondrial transfer therapies [25,61,188,189]. Post-treated patients could have periodic, noninvasive imaging monitoring to ensure that uncontrolled tumor growth does not arise. In addition, broader ethical considerations are related to the safety and efficacy of mitochondrial transfer in neurodegenerative diseases. Ensuring that mitochondrial transfer therapies are safe and effective for patients is crucial, and appropriate clinical trials and research must be conducted to address these ethical concerns [227]. To add to this complexity, obtaining informed consent from patients undergoing cell therapy is critical. Patients must fully understand the risks, benefits, and uncertainties associated with the treatment, including potential off-target effects and long-term implications. In the case of neurodegenerative diseases, patients may be vulnerable due to cognitive impairment, making it challenging to ensure genuinely informed consent [228]. In the context of vulnerable populations, and with any treatment, there is a risk of exploitation, particularly in patients located in regions with less stringent regulatory oversight [229]. Ethical guidelines must ensure that patients are not unduly influenced or coerced into participating in research or treatment protocols and are treated with dignity and respect [230].

Finally, the issue of equitable access in the context of mitochondrial medicine and cell therapy is a significant consideration [231,232]. As these innovative therapies continue to advance, addressing the potential disparities in access to these treatments is essential. Equitable access ensures that individuals from all socioeconomic backgrounds and geographic locations have the same opportunity to benefit from these medical advancements [233,234]. Barriers to equitable access may arise due to financial constraints, healthcare infrastructure, and awareness of these treatments. Existing cell therapy trials are overwhelmingly localized in high-income countries [230]. Mitochondrial medicine and cell therapy, being cutting-edge and often costly, may be out of reach for individuals with limited financial resources or those residing in underserved areas with inadequate healthcare facilities. Addressing the issue of equitable access requires concerted efforts from policymakers, healthcare providers, and researchers. Initiatives to reduce treatment costs, increase public awareness, and improve healthcare infrastructure in underserved areas are crucial for promoting equitable access to mitochondrial medicine and cell therapy. Developing sustainable funding models and reimbursement strategies can also help mitigate financial barriers, making these treatments more accessible to a broader population.

Overall, the ethical considerations in mitochondrial medicine revolve around patient safety, using stem cells, especially ESCs and iPSCs, and the potential implications of mitochondrial transfer; this requires careful assessment in developing and implementing these innovative therapies. Addressing the ethical concerns surrounding mitochondrial medicine necessitates a comprehensive and detailed approach that involves input from a wide range of stakeholders, including researchers, clinicians, ethicists, policymakers, and patient advocacy groups. Open and transparent dialogue among these stakeholders and a commitment to upholding moral principles are critical for navigating the intricate ethical considerations inherent in mitochondrial medicine, particularly in addressing neurodegenerative diseases. The well-being of patients must remain at the forefront of all decision-making processes in this complex medical landscape.

Ultimately, stem cell therapy relies on quality control standards to ensure the optimum outcomes for patients. Quality control screening of collected stem cells before use in a patient can be used to identify any genetic abnormalities that may predispose them to tumor formation [235,236,237]. The range of the standards includes contamination-free storage of an adequate number of stem cells in repositories obtained from the patient, which could be required if the patient develops a condition needing their stem cells [238,239,240]; screening of cells before use [238]; and noninvasive imaging to detect preexisting tumors before or when tumors arise after therapy [238].

Open and constructive collaborations among various groups, including government bodies, pharmaceutical companies, and nonprofit organizations, can play a pivotal role in expanding access to mitochondrial medicine and cell therapy. By working together, these entities can facilitate the development of inclusive healthcare policies, support research initiatives, and implement programs to ensure that individuals from diverse backgrounds can benefit from these transformative therapies.

## 6. Future Perspectives and Conclusions

Advancements in cell therapy hold immense promise for revolutionizing the treatment landscape of neurodegenerative diseases. Stem cell–based approaches, gene-modified therapies, and exosome-based strategies offer innovative solutions for combating disease progression, restoring mitochondrial function, and improving patient outcomes. In this context, focusing on mitochondrial biology and medicine is crucial for advancing our understanding of various diseases and developing effective treatment strategies. Mitochondria are vital organelles that generate energy to support critical cellular mechanisms, in addition to being central hubs for immune responses, neurotransmitter synthesis and recycling, and calcium buffering, among others. When these essential functions are compromised due to mitochondrial dysfunction, it can have far-reaching implications for human health. This dysfunction has been linked to various severe conditions, including neurodegenerative diseases such as Alzheimer’s and Parkinson’s, cardiovascular disorders, and metabolic disorders like diabetes. By exploring the intricate mechanisms of mitochondrial function and identifying potential interventions to restore or improve mitochondrial health, researchers and healthcare professionals can pave the way for innovative medical approaches that target the root causes of these conditions. Mitochondria health improvement must be considered contextually. In other words, interventions for Alzheimer’s may not be relevant for Parkinson’s (disease specificity). Likewise, cardiovascular or renal disease could rely on other treatment parameters (tissue specificity). For instance, amyloid-β in Alzheimer’s promotes mitochondrial anomalies, while alpha-synuclein creates mitochondrial dysfunction in Parkinson’s. Mitochondrial dysfunction contributes to kidney stones, whereas the mitochondria-associated endoplasmic reticulum membranes have a function that leads to cardiovascular diseases. 

To add to this complexity, understanding mitochondrial heterogeneity within a tissue is crucial for unraveling its implications for health and disease (e.g., [241,242,243]). In addition, mitochondrial heterogeneity within a cell refers to the differences in mitochondrial structure, function, and behavior within individual cells. These variations can arise due to several factors, including the cell’s metabolic state, location within the cell, and exposure to different environmental conditions. As highly dynamic organelles that constantly undergo fusion and fission processes, heterogeneous populations within a single cell arise. This dynamic nature allows mitochondria to adapt to the changing energy demands of the cell and respond to various stressors. The distribution of mitochondria within the cell can also contribute to heterogeneity (e.g., [244,245,246,247]). For example, mitochondria near the nucleus may experience different signaling cues and nutrient availability than those at the periphery or in dendritic processes [248,249].

Additionally, mitochondrial heterogeneity can arise from distinct mitochondrial subpopulations with specialized functions. Some mitochondria may be more involved in energy production, while others specialize in calcium buffering, reactive oxygen species (ROS) regulation, or involvement in cell death pathways (e.g., [250,251]). Perturbations in mitochondrial heterogeneity have been implicated in age-related decline, neurodegenerative disorders, metabolic diseases, and cancer. Therefore, elucidating the mechanisms governing mitochondrial diversity and its impact on cellular function holds promise for unveiling novel therapeutic targets and interventions to preserve mitochondrial health and tissue homeostasis. The overarching goal for each health disparity is finding the appropriate target. Relevant to neurological diseases, the alpha-synuclein in Parkinson’s and amyloid-β in Alzheimer’s have been heavily studied targets for therapeutic intervention, and the resulting benefit of target annihilation would also alleviate mitochondria dysfunction. Thus, solving mitochondria dysfunction in neurodegenerative diseases appears to rely on layers of complexities with different upstream mediators influenced by environmental, genetic, and gender-specific factors. While significant challenges lie ahead, current and continued research efforts and interdisciplinary collaboration for advancing the field of mitochondrial biology relevant to specific diseases and medicine are essential for realizing the full potential of cell therapy in addressing the unmet medical needs of individuals affected by neurodegenerative disorders.

## Figures and Tables

**Table 1:** Summary of selected studies identifying mitochondrial transfer as an essential mechanism in cell therapy. Many stem cell therapies for neurodegenerative diseases have not yet identified a mechanism for the treatment effects; however, mitochondrial transfer is likely one such mechanism.

Disease Phenotype	Method of Mitochondria Transfer	Tissue Type	Outcomes	References
Acute Respiratory Distress Syndrome	TNTs from MSCs	*in vivo* and *in vitro* alveolar macrophages	1. Increased macrophage phagocytosis function in receiving cells. 2. Blocking TNT formation reduced but did not eliminate mitochondrial transfer.	[27]
Asthma	TNTs from MSCs	*in vitro* human bronchial epithelial cells	1. Upregulation of Miro1 increases mitochondrial transfer rate. 2. Increased mitochondrial transfer reversed mitochondrial dysfunction and reduced asthma symptoms.	[30]
Alzheimer’s disease	EVs from MSCs	*in vitro* human neuronal cells	1. Reduced apoptosis, and mitochondrial dysfunction. 2. Reduced mitochondrial oxidative stress.	[31]
Parkinson’s disease	Direct injection of MSCs isolated mitochondria, peptide-mediated allogenic delivery	*in vivo* rat neuronal cells	1. Reduced dopaminergic neuron loss and improved mitochondrial dynamics leading to reduced ROS production.	[32]
Parkinson’s disease	Intranasal delivery of liver derived mitochondria	*in vivo* rat neuronal cells	1. Improved mitochondrial function and reduced oxidative stress.	[33]
Mitochondrial encephalomyopathy, lactic acidosis, and stroke-like episode	TNTs from MSCs	*in vitro* human neuronal cells	1. Restored mitochondrial function.	[34]
Parkinson’s disease	Undetermined transfer mechanism: not TNTs. Mitochondria were derived from iPSC-derived astrocytes	*in vitro* injured human dopaminergic neurons	1. Neurodegeneration was partially reversed due to mitochondria transfer. When mitochondria transfer was blocked but neurotrophic growth factors allowed, the cells showed no improvement. 2. iPSC-derived astrocytes can act as mitochondrial donors.	[35]

**Table 2:** Comparison of extracellular vesicles and MDV.

Vesicle Type	Size ^1^	Origin	Potential Cargo	Destination	References
Exosomes	30 to 140 nm	Inward budding of late endosomes and multivesicular body membrane or fusion with plasma membrane	RNA (primarily microRNA), mtDNA, mitochondrial proteins, transcription factors	Extracellular	[134,135,136,151]
MDVs	70 to 50 nm	Budding from mitochondrial membrane	Damaged or dysfunctional mitochondrial proteins	Lysosomes or cell membrane where cargo secreted in EV	[148,151,152,153]
Microvesicles	100 nm to 1 μm	Budding from plasma membrane	Intact mitochondria, DNA, RNA, ROS regulators, proteins, lipids	Extracellular	[154,155]

^1^ Mitochondria are approximately 0.5–1 μm [156].
